# Development and Validation of an Index to Measure the Quality of Facility-Based Labor and Delivery Care Processes in Sub-Saharan Africa

**DOI:** 10.1371/journal.pone.0129491

**Published:** 2015-06-24

**Authors:** Vandana Tripathi, Cynthia Stanton, Donna Strobino, Linda Bartlett

**Affiliations:** 1 Department of Population, Family Planning, and Reproductive Health, Johns Hopkins Bloomberg School of Public Health, Baltimore, Maryland, United States of America; 2 Department of International Health, Johns Hopkins Bloomberg School of Public Health, Baltimore, Maryland, United States of America; Shanghai 1st Maternity and Infant hospital of Tongji University, CHINA

## Abstract

**Background:**

High quality care is crucial in ensuring that women and newborns receive interventions that may prevent and treat birth-related complications. As facility deliveries increase in developing countries, there are concerns about service quality. Observation is the gold standard for clinical quality assessment, but existing observation-based measures of obstetric quality of care are lengthy and difficult to administer. There is a lack of consensus on quality indicators for routine intrapartum and immediate postpartum care, including essential newborn care. This study identified key dimensions of the quality of the process of intrapartum and immediate postpartum care (QoPIIPC) in facility deliveries and developed a quality assessment measure representing these dimensions.

**Methods and Findings:**

Global maternal and neonatal care experts identified key dimensions of QoPIIPC through a modified Delphi process. Experts also rated indicators of these dimensions from a comprehensive delivery observation checklist used in quality surveys in sub-Saharan African countries. Potential QoPIIPC indices were developed from combinations of highly-rated indicators. Face, content, and criterion validation of these indices was conducted using data from observations of 1,145 deliveries in Kenya, Madagascar, and Tanzania (including Zanzibar). A best-performing index was selected, composed of 20 indicators of intrapartum/immediate postpartum care, including essential newborn care. This index represented most dimensions of QoPIIPC and effectively discriminated between poorly and well-performed deliveries.

**Conclusions:**

As facility deliveries increase and the global community pays greater attention to the role of care quality in achieving further maternal and newborn mortality reduction, the QoPIIPC index may be a valuable measure. This index complements and addresses gaps in currently used quality assessment tools. Further evaluation of index usability and reliability is needed. The availability of a streamlined, comprehensive, and validated index may enable ongoing and efficient observation-based assessment of care quality during labor and delivery in sub-Saharan Africa, facilitating targeted quality improvement.

## Introduction

Global estimates show significant decreases in the number of maternal deaths in the past 15–25 years, with an estimated 289,000 deaths worldwide in 2013 [1]. However, only a minority of countries is on track to achieve the Millennium Development Goal (MDG) for reducing maternal mortality [1–3]. The lifetime risk of maternal mortality in sub-Saharan Africa remains 1 in 38 compared to 1 in 3,700 in developed countries [1]. Similarly, despite reductions in the past two decades, 3.3 to 3.6 million babies continue to die each year worldwide in the first month of life [4–5]. It is estimated that there are 2 million intrapartum stillbirths and intrapartum event-related early neonatal deaths each year [6].

Because maternal mortality is a rare event and many related indicators are difficult to measure at the population level, maternal health programs frequently evaluate progress through service utilization. The most common indicator is the skilled birth attendance rate, included in the maternal health MDG [7]. Facility delivery and, consequently, the use of a skilled birth attendant (SBA), are increasing in many developing countries, particularly where free delivery services or financial incentives have been introduced [8–9]. The presence of an SBA during delivery does not, however, guarantee the quality or content of care provided during labor & delivery (L&D) and the immediate postpartum period, including essential newborn care (ENC) [10–12].

Evidence has emerged from diverse settings that increasing facility delivery may not reduce mortality if quality of care (QoC) is poor [13–15]. There are also concerns about QoC in facilities facing increasing demand due to incentives for institutional delivery [14–16]. Past research indicates that coverage with effective interventions that may prevent or manage maternal and neonatal complications is low in many developing countries [17–20]. A 2013 analysis of WHO Multi-country Survey data suggests that coverage with life-saving interventions may be insufficient to reduce maternal deaths without overall improvements in the quality of maternal health care [21]. Improving QoC and increasing the provision of these interventions may decrease maternal and neonatal mortality [22–25].

Numerous criterion-based audits of maternal care suggest that failures in care processes are implicated in a substantial proportion of obstetric complications that result in death. Moreover, several studies in developing countries suggest that health system factors (e.g., failure to identify severity of condition, incomplete or inappropriate management, and lack of timely referral) contribute as much or more to severe maternal and perinatal morbidities or deaths as patient factors [26–29]. Miller et al., using record reviews, provider and patient interviews, and care observations, showed that poor quality of emergency obstetric and newborn care (EmONC) may underlie the persistence of high maternal mortality in the Dominican Republic despite high institutional delivery rates [13]. Other research suggests that inadequate QoC is implicated in low demand for facility-based L&D services and bypassing of closer facilities in favor of those that are more distant [30–32].

This body of evidence suggests that improving QoC is crucial in strengthening maternal and neonatal health. Maternity services, however, present unique challenges in quality assessment. The vast majority of deliveries are uncomplicated, but complications that do occur can result in serious morbidity or death for the mother and newborn [33]. Additionally, obstetric complications, such as postpartum hemorrhage (PPH), are unpredictable—they may occur in the presence of good, evidence-based clinical care, and may not occur in the absence of such care [22, 34]. These characteristics of maternity care make it difficult to assess QoC through clinical outcomes, particularly without large samples. It is also inadequate to base QoC assessment solely on provider knowledge or facility capacity to provide care; avoidable deaths may occur because available resources are not used [35].

The work of Avedis Donabedian provides a relevant framework, defining QoC by three components—structure, process, and outcomes [36–37]. Structure is readiness to provide care; process is actual service delivery; and outcomes are endpoints experienced by patients, related either to presenting health problems or care provided (e.g., iatrogenic infections) [36]. The process component may be the most useful to evaluate quality of maternal and newborn care (MNC), given the unpredictability of complications and relative rarity of maternal deaths. Salinas et al. found that process factors (provider and intervention) were associated with an 80-fold increase in risk of avoidable perinatal death, relative to an 11-fold increase due to structural factors (facility and context) [38].

QoC studies in sub-Saharan Africa have frequently assessed facility readiness to provide delivery services (structural quality) or evaluated outcomes using case fatality rates and similar indicators. Some research has also evaluated quality in terms of women’s satisfaction with health services, identified by Donabedian as a care outcome, without assessing technical QoC [39–40]. Many studies have been limited to documenting whether or not EmONC interventions are available or were recently provided, without assessing the quality of these services [41–42].

Measurement of the quality of the process of intrapartum and immediate postpartum care (QoPIIPC) is complex. Innovative tools and job aids have been developed to promote adherence to clinical guidelines and other aspects of process quality, such as the Safe Birth Checklist [43]. However, no standard consensus indicators exist to measure QoPIIPC in facilities in developing countries. A number of composite measures or summative checklists have been developed through expert opinion, but few have been validated; research suggests that empirical validation is important in strengthening quality measures [44].

While some studies have evaluated the technical aspects of care processes, for example through criterion-based audits, they have generally relied on retrospective analysis of incomplete data sources that were not intended to measure quality (e.g. maternity registers). Numerous studies have documented poor quality and limited sensitivity of obstetric facility records and databases for assessing the performance of care processes in both low- and high-resource settings [45–51].

Although observation is considered a gold standard in quality assessment, few studies have observed patient care to assess QoPIIPC. Service observation tools based on clinical guidelines are often lengthy, at times including hundreds of indicators [52–53], introducing the possibility of opportunities for measurement error. The large number of indicators also makes it difficult to assess QoPIIPC on an ongoing basis due to resource and time requirements.

Measurement of the quality of routine intrapartum and immediate postpartum services is essential in ensuring the delivery of appropriate interventions to reduce maternal and newborn mortality and morbidity. There is a need for valid and reliable measures as well as efficient tools to comprehensively measure QoPIIPC. Accordingly, this paper describes a study to identify the key dimensions of QoPIIPC in facility-based deliveries and to develop and evaluate a measure of these dimensions for application in sub-Saharan Africa. The study focused on the intrapartum and immediate postpartum periods when most maternal deaths occur and when care quality may have the greatest impact on both maternal and neonatal mortality [54–57]. It emphasized indicators of care processes for several reasons: the unpredictability of adverse maternal outcomes, the consequent difficulty of making inferences about QoC based on outcomes in small facilities or without adjusting for patient mix, and the importance of distinguishing the actual content of care from provider or facility capacity to provide care. Finally, the study targeted routine care—interventions or practices that should occur in all deliveries, rather than those that only apply to specific groups, for example multiparous women or neonates exhibiting danger signs.

## Methods

### Overview

Two data sources were used in this study. The first source was feedback and ratings obtained from global MNC experts. The second source was secondary data obtained from surveys observing L&D care at health facilities in sub-Saharan Africa.

The study began with a modified Delphi process using an MNC expert group to identify the important dimensions of QoPIIPC. Experts were also surveyed to determine which items from L&D care assessment tools characterize these dimensions and the importance of the items, regardless of dimension. Due to considerable heterogeneity in expert ratings, seven potential QoPIIPC indices were developed from combinations of highly rated items and preliminary analysis of L&D observation data. The seven indices were evaluated for face, content, and criterion validity, and a best performing index was selected. Face validity was assessed through expert feedback. Content and criterion validity were assessed across six domains, each with multiple benchmarks, using secondary data from L&D observations.

Because the description of methods includes terms with multiple meanings in different research and practice contexts, it is useful to define key concepts. In this study, dimensions are aspects of QoPIIPC that are distinct from each other but related to the larger quality construct, for example, interpersonal communication and respectful care. Items are discrete, observable actions that providers perform and that indicate QoPIIPC. Indices are combinations of items, summed to create a single score reflecting QoPIIPC. Face validity refers to whether likely users, such as MNC experts and care providers, perceive an index to include important, feasible, and appropriate items for assessing QoPIIPC in sub-Saharan Africa. Content validity refers to whether an index represents all key dimensions of QoPIIPC, assessing the full range of important aspects of care. Criterion validity refers to the association of the index score with a related measure of QoPIIPC considered to be a reasonable standard for assessing this construct. In this case, the related measure is performance on a comprehensive L&D care assessment tool, described below. The ability of a shorter index of items to serve as a proxy for performance on the comprehensive tool reflects its criterion validity.

In this study, the term validation domains refers to topics evaluating the degree to which an index measures and is informative about QoPIIPC. Benchmarks are specific, quantifiable, and comparable criteria within each validation domain.

### Identification of the key dimensions of QoPIIPC

An *a priori* model of the dimensions of QoPIIPC was developed based on the Donabedian QoC framework, relevant theory, and empirical evidence. This model included three dimensions: 1) use of evidence-based interventions adhering to global guidelines (technical quality); 2) inter-personal communication and respectful care (interpersonal quality); and 3) ongoing monitoring of the patient and watchful supervision throughout the intrapartum and IP periods (monitoring quality).

Consensus on the dimensions of L&D QoC was developed through a modified version of the Delphi process, conducted with a group of U.S.-based experts in global MNC. Modifications to the original Delphi process developed by the RAND Corporation included the use of an in-person meeting and changes to the scoring systems [58–59]. Items assessing routine care from a structured L&D observation checklist applied in a series of maternal and newborn QoC health facility surveys were used to facilitate consensus building. The surveys, known as the QoC Assessments, were conducted by the Maternal and Child Health Integrated Program (MCHIP), a USAID-funded global maternal and child health project directed by Jhpiego. The surveys are described further below and S1 Checklist provides the full QoC Assessment L&D observation checklist.

**Fig 1:**
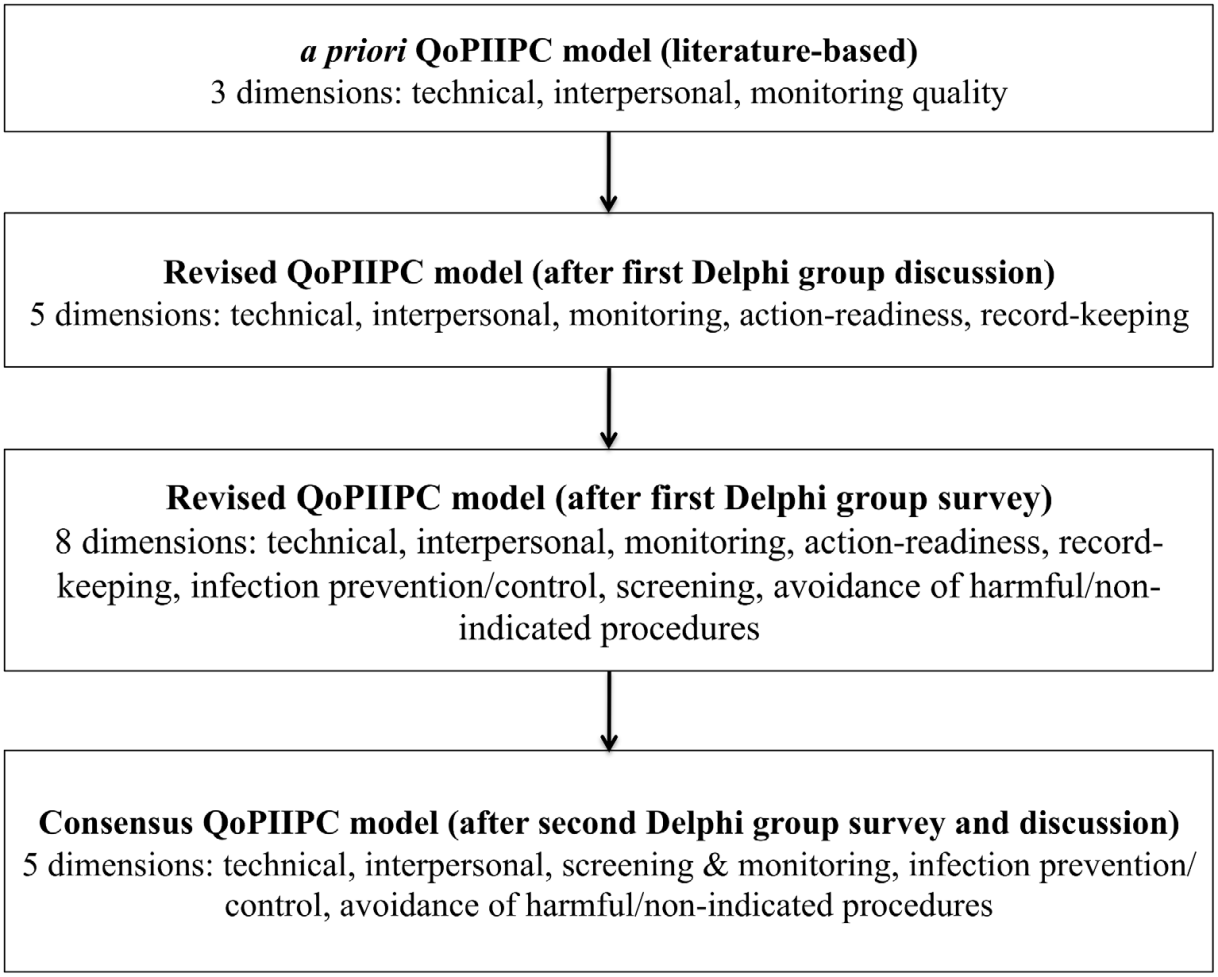
Delphi process to identify the key dimensions of QoPIIPC

Fig 1 describes the iterative development of a consensus model of QoPIIPC. At the start of this process, the MNC expert group met to discuss potential dimensions of QoPIIPC. Experts worked in small groups to categorize a sample of 15 items from the QoC Assessment L&D observation checklist into potential dimensions of QoPIIPC. The sample represented provider actions undertaken throughout the intrapartum and immediate postpartum periods, including ENC. The full group re-convened and discussed dimensions into which items could be grouped. The *a priori* model of QoPIIPC was revised based on expert feedback.

The experts were then surveyed to rate the 131 routine care items from the QoC Assessment L&D observation checklist, scoring each item on two characteristics: how well it represented each potential dimension of QoPIIPC and its overall importance, irrespective of dimension. Dimension representation was rated from 1, meaning that the item was not representative at all of a specific dimension, to 3, meaning that the item was very representative. Overall importance was rated from 1, meaning that the item was unimportant, to 4, meaning that it was essential. Mean ratings were calculated for each item. Experts also provided qualitative comments regarding dimensions and items. Because the QoC Assessment L&D observation checklist was based on WHO guidelines and the work of clinical and research experts, it is presumed to provide a comprehensive, evidence-based item pool for tasks, interventions, and procedures that indicate QoPIIPC. Further information about the sources of these items and the evidence for their importance is provided below. At the recommendation of the original expert group, this survey was also administered to MNC experts based in sub-Saharan Africa and at global health institutions, separately from the consensus process on QoPIIPC dimensions.

Following discussion of the results of the first survey, a second survey was undertaken. Experts rated each proposed dimension on its importance, with 1 being not important and 3 being essential; and its distinctness from other dimensions, with 1 being not distinct/mostly overlapping with other dimensions and 3, very distinct/mostly unique from other dimensions. Based on expert group ratings, a consensus model of QoPIIPC was defined. This model was applied in subsequent analysis to develop a measure of QoPIIPC.

The surveys used to identify consensus QoPIIPC dimensions and rate potential index items are described in Table 1. In all, 32 individuals participated in consensus development meetings and and/or item rating surveys.

**Table 1:** Expert surveys and respondent groups.

****Survey****	****Respondent category (n)****
1) Rating all routine care items in the QoC Assessment delivery observation checklist on a) overall importance and b) ability to represent each proposed dimension of QoPIIP	U.S.-based MNC expert group (7)
2) Rating each proposed dimension of QoPIIPC on a) importance and b) uniqueness	U.S. based MNC expert group (7)
3) Rating all routine care items in the QoC Assessment delivery observation checklist on a) overall importance and b) ability to represent each proposed dimension of QoPIIPC	Expanded global/regional MNC expert group (16)

### Development of a measure of QoPIIPC

#### Secondary data source

Secondary data were drawn from the MCHIP QoC Assessments. These surveys have been carried out in Bangladesh, Ethiopia, Kenya, Madagascar, Malawi, Mozambique, Pakistan, Rwanda, Tanzania, Zanzibar, and Zimbabwe. Initial QoC Assessments were conducted in 2010–2012, with repeat surveys in Tanzania and Zanzibar in 2012–2013. This study used data from Kenya, Madagascar, both rounds in Tanzania, and the first round in Zanzibar, based on data availability at the time of analysis and comparable maternal health and services indicators in these countries [60–62].

The structured checklists used for clinical observation in the QoC Assessment surveys provided a comprehensive list of important actions during L&D care, informed by research evidence, clinical guidelines, and programmatic experience. The checklists were based on protocols in the World Health Organization (WHO) manuals for the Integrated Management of Pregnancy and Childbirth and for Managing Complications in Pregnancy and Childbirth and drew on item sources such as the ACCESS Project’s *Best Practices in Maternal and Newborn Care*: *Learning Resource Package;* Stanton *et al*.*’s* international survey of the active management of the third stage of labor (AMTSL), conducted through the Prevention of Postpartum Hemorrhage Initiative (POPPHI); and Jhpiego’s checklists for trainers to observe simulated basic EmONC practices by trainees [11, 15, 33, 63–64]. Checklist items were also selected based on the major causes of maternal and neonatal mortality and reviewed by the study team.

The initial observation checklists resulting from this process were reviewed by a larger group of trainers, clinicians, and monitoring and evaluation experts. The draft observation instruments and training techniques were then field-tested as stand-alone tools in Ethiopia and embedded within the more comprehensive Service Provision Assessment (SPA) in Kenya. In both settings, national experts, trainers, and trainees who were all expert facility assessors or clinicians provided feedback on the tools in advance and after data collection. After these pilots, a final core set of QoC survey observation checklists was created. These core tools were also pre-tested in every country where the QoC survey was implemented.

The final routine care checklist used in the MCHIP QoC surveys included items on essential L&D care, such as partograph use, infection prevention, client-provider interactions, AMTSL, and immediate newborn care. Additional checklists contained items on management of complications, including postpartum hemorrhage (PPH), pre-eclampsia or eclampsia (PE/E), and newborn resuscitation [65]. As indicated by the source documents above, the 131 items in the routine care checklist (S1 Checklist) reflect evidence-informed guidelines and current global consensus on best practices.

The MCHIP surveys also collected information about health facilities and providers and some background maternal characteristics [65]. While tools were revised between the first and second surveys, most variables are identical or have equivalents across QoC Assessments.

Data were collected by personnel trained specifically for the QoC Assessments. Observers were already trained in MNC clinical skills. An 11-day training curriculum for data collectors included clinical updates, review and mastery of the content of the data collection instruments and procedures for informed consent and confidentiality, and practice to ensure the validity and reliability of clinical observation [66–68]. Classroom training included exercises and practice until inter-rater reliability for clinical observations was established at a level of 80% agreement or higher. Trainees observed the practice of key interventions on anatomic models using “flawed” clinical performances; their scores were compared to the correct responses as determined by the designers of the training exercises. These exercises were followed by group discussion about observing and recording clinical data. Trainees then spent two days observing deliveries and practicing using the data collection tools at health facilities. In most countries, data were collected on smartphones with customized data entry applications. Data from each device was uploaded directly to a central online database or a secure digital card each day [66–67]. In Kenya the QoC Assessment was nested within the 2010 Service Provision Assessment (KSPA) Survey [69]. The KSPA used paper-based surveys and its own data collection team. However, additional observers were recruited, supported, and trained by MCHIP for L&D observation. Similar training procedures were followed in Kenya as in other countries [68–69].

The QoC Assessments were designed to provide national estimates of routine facility-based L&D practices, with samples of at least 250 deliveries in each country. Each country’s sampling plan was adapted to meet local needs. In Kenya the survey is nationally representative. In Madagascar the survey covered all facilities with two or more deliveries per day. In Tanzania and Zanzibar the survey sampled facilities in regions participating in quality improvement projects. At each sampled health facility, all births (or as many as were feasible for data collectors to observe) were to be sampled at each facility during the survey period, which was generally two days per facility [66–68].

Additional information about survey development, sampling, and data collection procedures is available in country QoC Assessment reports and reported in publications describing key findings in Madagascar and Tanzania [53, 65–70].

#### Exploratory analyses of survey data

Variables differing across country datasets were modified as needed. Due to small numbers of deliveries observed in Zanzibar in the first survey round, the sample was combined with the Round 1 Tanzania sample for analysis. Unweighted data were used for analysis. Proportions or means (with 95% confidence intervals) were calculated for all variables along with graphical exploration of data distributions. χ2 tests and t-tests were used to identify differences across variables between and within countries.

Because the study sought to develop a comprehensive QoPIIPC measure, data analysis was restricted to L&D cases observed across initial intake, active labor, delivery, and the immediate postpartum period. The complete survey samples and samples included in analysis were compared to identify any differences in available background variables (e.g., maternal characteristics).

#### Development and validation of QoPIIPC indices

The modified Delphi process was used to reach consensus regarding key dimensions of QoPIIPC. However, review of expert survey responses showed substantial variation among different expert subgroups in the ratings of specific items to represent these dimensions. Therefore, rather than selecting a QoPIIPC index through a consensus or Delphi process, seven possible QoPIIPC indices containing combinations of highly rated items were evaluated according to specified validation domains, described below. The indices ranged in length from 17 to 23 items. Index A, the preliminary QoPIIPC index, contained items rated highly by the original US-based MNC expert group participating in the modified Delphi process. Index B, referred to as the “3+ index”, contained items rated highest by at least three of the four surveyed groups (all experts, participants in the Delphi process, experts in sub-Saharan Africa, and experts at global health institutions). Index C contained the items with the highest mean importance ratings across all experts. Index D contained items with the highest mean ratings across global MNC experts. Finally, Index E contained items with the highest mean ratings across regional experts in sub-Saharan Africa.

Two indices were constructed using additional information: qualitative feedback from MNC experts and exploratory analysis of QoC Assessment survey data. The first constructed index (Index F) combined the 3+ index with additional items considered to improve content validity based on expert feedback, including two newborn care items and an item reflecting interpersonal care. The second constructed index (Index G) built on the first, but omitted 3 items that were nearly universally performed or considered by the expert group to be difficult to observe and, therefore less informative about care quality. The omitted items were replaced with others considered easier to observe or performed less frequently.

#### QoPIIPC scores

Country data from QoC Assessments in Kenya, Madagascar, Tanzania, and Zanzibar were used to validate the potential QoPIIPC indices. Each observed, eligible delivery was assigned index scores based on performance of items in each index. An item was assigned a value of 1 if it was performed, and 0, otherwise. Item scores were summed to create QoPIIPC index scores for each delivery. Each delivery observation was also assigned a total QoC performance score by summing performance of all routine care items in the full QoC Assessment delivery checklist.

#### Validation domains and benchmarks

Six content and criterion validation domains were used to assess the QoPIIPC indices: the degree to which an index included items representing all key QoPIIPC dimensions, association of the index score with the total QoC performance score, association of each item in an index with the total QoC performance score, ability of an index to discriminate between poorly and well-performed deliveries, inclusion of items in an index across a range of performance frequency (from rarely to frequently performed), and the variability and distribution of the index score. Benchmarks and selection criteria for each domain are described in Table 2. A threshold of p = 0.05 was used for all benchmarks that included assessment of statistical significance. In addition to stand-alone assessments of each index, comparative analyses treated the preliminary QoPIIPC index (Index A) and the 3+ index (Index B) as reference models.

**Table 2:** Index validation domains, benchmarks, and selection criteria.

Validation domain	Number of benchmarks	Description of benchmarks^1^	Selection criteria by benchmark
1. Representation of QoPIIPC dimensions	2	a. Number of QoPIIPC dimensions represented (1)	a. Highest number of dimensions represented
		b. Balance among QoPIIPC dimensions (1)	b. Smallest difference in the number of items from each represented dimension
2. Association of index score with overall QoC performance	3	a. Linear regressions of total QoC scores against index scores (1)	a. Largest magnitude of standardized regression coefficient, significant p-value, lowest AIC/BIC, lowest RMSEA, highest R2
		b. Likelihood ratio tests (LRTs) comparing indices to the 3+ index (1)	b. Significant p-value (<0.05) for χ2 test
3. Item association with overall QoC performance	3	a. Number of items with no statistically significant association with total QoC score (1)	a. Lowest number of items without association with total QoC scores
		b. Number of items with no statistically significant association with odds of relative or absolute good performance^2^ (2)	b. Lowest number of items without association with total QoC scores
4. Ability to discriminate poorly and well-performed deliveries	15	a. Logistic regressions of good and poor performance categories of total QOC score against index scores^3^ (3)	a. Largest magnitude of standardized odds ratio, significant p-value, lowest AIC/BIC, highest Efron’s R2
		b. LRTs comparing performance of indices on logistic regressions, with 3+ index as reference model (3)	b. Significant p-value (<0.05) for LRT chi2 test
		c. AUROCs of indices’ ability to discriminate poorly and well performed deliveries, based on logistic regressions (3)	c. Largest AUROC
		d. Comparison of AUROCs against both reference indices^4^ (6)	d. Significant p-value for AUROC comparison **χ**2 test
5. Inclusion of items across a range of performance frequency (from rarely to frequently performed)	3	a. Number of items performed in <30% of observed deliveries (1)	a. Highest number of items performed in <30% of deliveries
		b. Number of items performed in <40% of observed deliveries (1)	b. Highest number of items performed in <40% of deliveries
		c. Number of items performed in >90% of observed deliveries (1)	c. Lowest number of items performed in >90% of deliveries
6. Variability and distribution of index score	3	a. Coefficient of variation (CoV) (1)	a. Largest CoV
		b. % of deliveries receiving the minimum QoPIIPC index score (1)	b. ≤15% of deliveries with minimum score
		c. % of deliveries receiving the maximum QoPIIPC index score (1)	c. ≤15% of deliveries with maximum score

^1^Numbers in parentheses refer to number of analyses done for each benchmark

^2^Relative good performance (top 25% of distribution), absolute good performance (≥80% of items correct), and relative poor performance (bottom 25% of distribution)

^3^Three separate simple logistic regression models, using the three dichotomous variables created to represent the total QoC score (see footnote 2)

^4^The reference models are the 3+ index and the preliminary QoPIIPC index.

Ability to discriminate between poorly and well-performed deliveries is a key attribute of a meaningful quality measure for L&D care. Accordingly, this domain accounts for the majority of validation benchmarks. Analyses associated with this domain are described further here.

To enable assessment of QoC discrimination, level of overall care quality was evaluated by three dichotomous variables. First, relative good performance was defined as being in the top 25% of the total QoC score distribution. Second, absolute good performance was defined as achieving at least 80% of the maximum possible total QoC score. Third, relative poor performance was defined as being in the bottom 25% of the total QoC score distribution. The three variables were treated as dependent variables in separate analyses. A series of simple logistical regressions estimated the relation between the index score and the odds of good or poor performance. Model fit for each index was compared to the two reference indices (Indices A and B) through likelihood ratio tests.

The area under the receiver operating characteristic (AUROC) curves based on the logistic regression results was assessed for each good/poor performance group. The AUROC indicates the ability of an index to correctly classify QoC: if two deliveries are drawn from the sample at random, the AUROC indicates the proportion of pairs in which the delivery with the higher QoPIIPC index score is in the good performance group (and vice versa, for poor performance). An AUROC of 0.7 to 0.9 is considered to show moderate discrimination, while over 0.9 shows excellent discrimination [71–72]. AUROCs were also compared to assess the ability of each index to classify deliveries as poorly or well-performed relative to the reference indices.

#### Index scoring and selection

Indices were evaluated separately using delivery data for each country. Comparisons involved creating and summing validation performance scores at the benchmark, domain, and country level. The scoring system is described in Table 3. The best performing index was selected based on the validation performance scores within each country and across countries. For the selected QoPIIPC index, predicted probabilities of being in the good performance groups of the total QoC score were calculated for each value of the index score based on logistic regressions.

**Table 3:** Steps in scoring QoPIIPC indices and comparing validation performance.

Benchmark level	For each validation benchmark (see Table 2), the index that performed best received a value of 2; the next best performing index received a value of 1. All other ranks received a value of zero, and ties were acceptable. The top two indices were ranked to avoid selection of one top performer based on small differences.
Domain level	Performance on each benchmark (ranging from zero to 2) was summed within each of the six validation domains. 2 points were assigned to the index with the highest score in each domain, and 1 point was assigned for the second-highest score.
Country level	Domain scores were summed into validation performance scores for each index within each country. These validation performance scores ranged from 0 to 12 (up to 2 points per domain). Validation performance scores were also summed across countries for each index.
Index selection	The best performing index was selected based on these validation performance scores, both within each country and across all countries.
Sensitivity analysis	The primary scoring approach was designed to provide equal weight to each validation domain in index selection. Each domain, however, had a different number of validation benchmarks. More benchmarks were related to the index ability to discriminate poorly and well-performed deliveries than to other domains (15 out of 28). The scoring system, therefore, results in less weight assigned to each benchmark in domains with more benchmarks. An alternative score summation approach was also used to evaluate whether a different index would be selected if all benchmarks were given equal weight. In this alternative approach, a total validation performance score was summed directly across all benchmarks and across all countries, without first summing and ranking with each validation domain.

### Ethical considerations

The protocol was reviewed and approved by ethical review boards in each country where the QoC Assessment was conducted. In the countries whose data are analyzed in this study, these boards were: the Kenya Medical Research Institute Institutional Review Board (IRB) in Kenya; the Ministry of Health Ethical Committee in Madagascar; and the National Institute of Medical Research Institutional Review Board IRB in Tanzania. The Johns Hopkins Bloomberg School of Public Health (JHSPH) IRB ruled the protocol exempt from review. During the QoC Assessments, written informed consent was obtained from facility directors, along with verbal informed consent of the providers and patients or patients’ next of kin. Written consent was not obtained from providers because they were encountered during the process of providing L&D care and an extensive discussion of benefits and burdens had been conducted with facility directors in a non-service provision context. Written consent was not obtained from patients or next of kin both because of literacy limitations and to reduce the burden on women being encountered during L&D. Data collection team supervisors obtained facility-level written consent forms. Verbal consent was recorded in the QoC Assessment data entry applications, with each module of questions noting that provider and patient (or next of kin) consent must be obtained before items in that module could be completed. Consent procedures were described in research plans submitted to and approved by the aforementioned IRBs. The names of individual patients were not collected during service observations. Quantitative analyses were conducted using secondary data without patient identifiers.

## Results

### Key dimensions of QoPIIPC

The Delphi process results are shown in Fig 1. Initial feedback resulted in five proposed dimensions of QoPIIPC. Two dimensions proposed in addition to those initially hypothesized (technical, interpersonal, and monitoring QoC) were: action-readiness, providers’ preparation and ability to respond to signs of complication and action cues; and record-keeping, the completion of administrative and medical records. For technical QoC, 113 of the 131 routine L&D care items were deemed somewhat or very representative, suggesting that this concept was too broad to be a meaningful QoC dimension. Qualitative comments also suggested that three additional dimensions could be isolated from technical QoC: infection prevention/control, screening for danger signs, and avoidance of harmful/non-indicated procedures. All 8 potential dimensions were included in the second expert survey (see Table 1). S1 Table summarizes the findings of this survey.

Ultimately, five dimensions were retained in the consensus QoPIIPC model: technical, interpersonal care, screening & monitoring, infection prevention/control, and avoidance of harmful/non-indicated interventions. Table 4 summarizes this model and provides an example item for each dimension. The consensus dimensions were applied in assessment of the content validity of potential QoPIIPC indices.

**Table 4:** Consensus model of QoPIIPC and sample items.

Dimension	Sample item
Technical quality	Ties or clamps cord when pulsations stop, or by 2–3 minutes after birth (not immediately after birth)
Interpersonal	At least once, explains what will happen in labor to the woman and/or her support person
Screening & monitoring	Takes mother's vital signs 15 minutes after birth
Infection prevention/control	Washes his/her hand before any examination
Avoidance of harmful/non-indicated practices	Was there use of episiotomy without appropriate indication?

### Development of a measure of QoPIIPC

Table 5 provides sample sizes for the full QoC Assessment for each country and the number of deliveries included in the analysis. About two-thirds of deliveries in Kenya and Madagascar were retained in the analysis. More women arrived later in the L&D process in facilities in Tanzania and Zanzibar, resulting in the inclusion of 39–40% of observed deliveries in the analysis. An examination of available background characteristics (facility type; provider category; and mother’s HIV status, gravidity, and parity) found that there were almost no significant differences between the full QoC Assessment samples and the deliveries included in analysis (see Table 6) The analysis sub-sample in Tanzania Round 2 had fewer hospital deliveries and more health center deliveries.

**Table 5:** QoC Assessment sample sizes.

Country	Facilities	Deliveries—full sample	Deliveries—included in analysis^1^ (%)
Tanzania (incl. Zanzibar) Round 1	56	706	282 (39.9%)
Tanzania Round 2	48	558	220 (39.4%)
Kenya	170	626	403 (64.4%)
Madagascar	36	347	210 (60.5%)
**Total**	**506**	**2,238**	**1,145 (51.2%)**

^1^Deliveries were included in analysis if observed at intake, during labor and delivery, and immediately postpartum.

**Table 6:** Characteristics of observed deliveries^1^.

Characteristic	Full sample	Included in analysis	Comparison (t-test and χ^2)^
**Kenya**			
	**n = 626**	**n = 403**	
Mean gravidity of mother^2^	N/A	N/A	N/A
Mean parity of mother^2^	N/A	N/A	N/A
*HIV status* ^2^	N/A	N/A	N/A
*Facility type*			0.978
National referral hospital	4.79%	5.71%	
Provincial hospital	13.58%	12.90%	
District hospital	39.62%	41.44%	
Sub-district or other hospital	35.94%	34.74%	
Health center	3.67%	2.98%	
Dispensary	0.32%	0.25%	
Maternity	2.08%	1.99%	
*Provider category*			0.967
Doctor	0.96%	0.74%	
Nurse	90.10%	89.33%	
Midwife	8.31%	9.43%	
Other	0.64%	0.50%	
**Madagascar**			
	**n = 347**	**n = 210**	
Mean gravidity of mother	2.40	2.40	0.994
Mean parity of mother	2.15	2.10	0.755
*HIV status*			0.320
Positive	0.57%	0.95%	
Negative	62.36%	70.00%	
Unknown	35.06%	29.05%	
*Facility type*			0.680
University hospital	35.06%	37.62%	
Regional hospital	24.43%	20.48%	
District hospital 1	6.61%	7.14%	
District hospital 2	14.66%	12.38%	
Basic health center 2	19.25%	22.38%	
*Provider category*			0.825
Doctor	18.84%	16.27%	
Nurse	3.77%	4.31%	
Midwife	69.28%	69.86%	
Other	8.12%	9.57%	
**Tanzania (including Zanzibar) Round 1**			
	**n = 706**	**n = 282**	
Mean gravidity of mother	3.08	2.84	0.950
Mean parity of mother	2.15	1.94	0.072
*HIV status*			0.931
Positive	4.45%	3.99%	
Negative	93.24%	93.48%	
Unknown	2.30%	2.54%	
*Facility type*			0.611
Regional hospital	27.62%	27.66%	
Hospital	29.32%	25.89%	
Health center	40.80%	43.26%	
Dispensary	2.27%	3.19%	
*Provider category*			0.917
Doctor	1.85%	1.42%	
Nurse	32.76%	34.52%	
Midwife	55.70%	54.09%	
Other	9.69%	9.96%	
**Tanzania (Round 2)**			
	**n = 558**	**n = 220**	
Mean gravidity of mother	2.58	2.61	0.810
Mean parity of mother	1.71	1.66	0.936
*HIV status*			0.508
Positive	91.81%	6.51%	
Negative	4.48%	90.23%	
Unknown	3.70%	3.26%	
*Provider category*			0.996
Doctor	2.34%	1.82%	
Nurse	46.76%	45.91%	
Midwife	49.46%	50.45%	
Other	1.44%	1.82%	
*Facility type*			0.020
Regional hospital	0.54%	0.00%	
Hospital	61.11%	52.28%	
Health center	31.36%	42.48%	
Dispensary	6.99%	5.45%	

^1^Missing observations are excluded; <10% of observations in each sample; Yates correction applied due to low cell frequencies

^2^These questions were not included in the delivery observation module in the Kenya survey.

Table 7 describes the seven potential QoPIIPC indices evaluated. Table 8 lists the items in each index. Overall, 33 items were included across indices, with a high number of overlapping items. S2 Table describes the proportion of deliveries in which these items were performed in each country. While many processes assessed by survey items were performed at similar frequencies across countries, there was notable variation, particularly increases in performance of several items between the first and second surveys in Tanzania.

**Table 7:** Description of 7 potential QoPIIPC indices evaluated across validation benchmarks.

Potential index	Description	Number of items
A. Preliminary index^1^	Items with a mean importance rating of ≥3.75 out of 4; rated by original MNC expert group.	20
B. 3+ index^1^	Items rated highly by 3 or more expert subgroups.	17
C. All-survey index	Items with a mean importance rating of ≥3.5 out of 4; rated by all surveyed experts.	21
D. Global index	Items with a mean importance rating of ≥3.6 out of 4; rated by all experts based at global health institutions.	23
E. Africa region index	Items with a mean importance rating of ≥3.8 out of 4; rated by experts based in sub-Saharan Africa.	21
F. Constructed index^1^	3+ group plus an additional 3 items recommended by MNC experts during initial face validity assessment.	20
G. Constructed index^2^	Adapted from constructed index 1. Removed 3 items that were universally performed or identified by expert group as difficult to observe accurately. Replaced with 3 items that were less frequently performed and/or recommended during expert group feedback.	20

^1^Indicates that this index served as a reference model in comparisons of AUROCs and/or likelihood ratio tests.

**Table 8:** Items included in potential QoPIIPC indices^1^.

	Potential QoC indices						
Item	A	B	C	D	E	F	G
Checks woman's HIV status (checks chart or asks woman) and/or offers woman HIV test	+	+	+	+	+	+	+
Asks whether woman has experienced fever	-	-	-	-	+	-	-
Asks whether woman has experienced convulsions or loss of consciousness	-	-	-	-	+	-	-
Asks whether has experienced headaches or blurred vision	-	-	-	-	+	-	+
Asks whether woman has experienced vaginal bleeding	-	+	+	+	+	+	+
Takes temperature	+	-	-	+	-	-	-
Takes pulse	+	+	+	+	-	+	+
Takes blood pressure	+	+	+	+	-	+	+
Tests urine for presence of protein	+	-	-	-	-	-	-
Washes his/her hand before any examination (initial & during labor)	+	+	+	+	+	+	+
Abdominal examination (fetal presentation & fetal heart rate)	+	+	+	+	+	+	-
Vaginal examination (cervical dilation; fetal descent, position, membranes, meconium)	+	+	+	+	+	+	-
Wears high-level disinfected or sterile gloves for vaginal examination	+	+	+	+	+	+	+
At least once, explains what will happen in labor to the woman and/or her support person	+	-	-	+	-	+	+
Uses partograph	+	+	+	+	+	+	+
Prepares uterotonic drug to use for AMTSL	+	+	+	+	+	+	+
Self-inflating ventilation bag (500mL) and face masks (size 0 and size 1) are laid out and ready for use for neonatal resuscitation*	+	+	+	+	+	+	+
At least 3 cloths/blankets (1 to dry, 1 to cover, 1 to elevate shoulders) are laid out and ready for use for neonatal resuscitation	+	-	-	-	-	-	-
Puts on clean protective clothing in preparation for birth that protects face, hands, and body from contact with body fluids	-	-	-	-	+	-	-
As baby's head is delivered, supports perineum	+	-	-	-	-	-	-
Correctly administers uterotonic (timing, dose, route)	+	+	+	+	+	+	+
Performs uterine massage immediately after delivery of placenta	-	-	-	-	+	-	-
Assesses for perineal and vaginal lacerations	-	+	+	+	+	+	+
Assesses completeness of placenta and membranes	-	+	+	+	+	+	+
Immediately dries baby with towel	+	+	+	+	+	+	+
Discards wet towel and covers with dry towel	-	-	+	-	+	-	-
Places newborn on mother’s abdomen skin-to-skin	-	-	-	-	+	-	+
Ties or clamps cord when pulsations stop, or by 2–3 minutes after birth	+	-	-	+	-	+	+
Cuts cord with clean blade	+	+	+	+	+	+	-
Disposes of all sharps in puncture-proof container immediately after use	-	-	+	+	-	-	-
Takes mother's vital signs 15 minutes after birth	+	+	+	+	-	+	+
Palpates uterus 15 minutes after delivery of placenta	-	-	+	+	-	-	+
Assists mother to initiate breastfeeding within one hour	-	-	+	+	-	+	+

^1^Items are provided in the order in which they are expected to be performed over the course of an episode of labor and delivery care.

Summary scores for index performance across the validation domains, based on the scoring system described in Table 3, are presented in Table 9. While all indices performed reasonably well, three appeared to be the most informative about QoPIIPC. They were the index containing items ranked most highly by the sub-Saharan African regional expert group (Index E) and the two indices (Indices F and G) constructed based on expert group rating combined with information from exploratory analysis of survey data and qualitative feedback from MNC experts. While Index E performed well in terms of validation benchmarks, there were concerns about its content validity, such as a lack of items for the immediate postpartum period. Table 10 shows the performance of the constructed indices alongside the two reference models, the preliminary QoPIIPC index (Index A) and the 3+ index (Index B).

**Table 9:** Summary of index performance across validation domains^1^.

	Potential QoC indices						
	A	B	C	D	E	F	G
**Kenya**							
Dimension representation	0	0	0	2	0	1	0
Association with overall QoC	0	0	0	0	0	2	2
Discrimination of good/poor performance	0	0	0	0	0	2	1
Item association with overall QoC	2	2	0	0	2	2	1
Item performance range	1	0	0	0	0	0	2
Variability and distribution of index score	0	1	0	0	0	0	2
**Total**	**3**	**3**	**0**	**2**	**2**	**7**	**8**
**Madagascar**							
Dimension representation	0	0	0	2	0	1	0
Association with overall QoC	0	0	0	0	2	0	2
Discrimination of good/poor performance	0	0	0	0	1	0	2
Item association with overall QoC	0	1	0	0	2	0	0
Item performance range	0	0	0	0	2	0	1
Variability and distribution of index score	0	0	0	0	1	0	2
**Total**	**0**	**1**	**0**	**2**	**8**	**1**	**7**
**Tanzania R1 (incl. Zanzibar)**							
Dimension representation	0	0	0	2	0	1	0
Association with overall QoC	0	0	0	1	0	1	2
Discrimination of good/poor performance	0	0	0	0	1	0	2
Item association with overall QoC	1	1	0	1	0	1	2
Item performance range	0	0	0	0	1	0	2
Variability and distribution of index score	1	0	0	0	0	0	2
**Total**	**2**	**1**	**0**	**4**	**2**	**3**	**10**
**Tanzania R2**							
Dimension representation	0	0	0	2	0	1	0
Association with overall QoC	0	0	0	0	2	0	1
Discrimination of good/poor performance	0	0	0	0	2	0	2
Item association with overall QoC	0	2	1	0	0	0	2
Item performance range	2	0	0	0	0	0	1
Variability and distribution of index score	0	0	0	0	2	0	1
**Total**	**2**	**2**	**1**	**2**	**6**	**1**	**7**
**Total across countries**	**7**	**7**	**1**	**10**	**18**	**12**	**32**

^1^Each index received 2 points if it was the best performing on the measures of a particular benchmark, and 1 point if it was the second best performing. All other ranks received 0 points and ties were acceptable. The scoring system is described in Table 3.

**Table 10:** Comparison of reference and constructed QoPIIPC indices^1^ —Descriptive statistics and performance on benchmarks across validation domains using Tanzania (incl. Zanzibar) Round 1 data.

	Index A: Preliminary QoPIIPC Index	Index B: 3+ Index	Index F: Constructed Index 1	Index G: Constructed Index 2
**Descriptive statistics**				
Mean (% of maximum achievable)	13.52 (64.38%)	12.43 (69.06%)	14.11 (67.19%)	12.12 (57.71%)
High score (% of maximum achievable)	21 (100.00%)	18 (100.00%)	21 (100.00%)	21 (100.00%)
Low score (% of maximum achievable)	1 (4.76%)	1 (5.56%)	1 (4.76%)	0 (0.00%)
**Validation Domain**				
**- benchmark(s)**				
Representation of QoPIIPC dimensions				
- # of dimensions (out of 5)	4	3	4	4
Association of index with overall QoC performance				
- B coefficient from SLR of total QoC score (p-value)	8.35^2^ (<0.001)	8.32 (<0.001)	8.812 (<0.001)	8.92^2^ (<0.001)
Association of individual items with overall QoC performance				
- # of items without significant relationship to total QoC score	3	3	3	1
- # of items without significant relationship to good total QoC score (absolute)	5	5	5	4
- # of items without significant relationship to good total QoC score (relative)	3	3	3	3
Ability to distinguish between good and poor performance				
- AUROC good total QoC score—absolute	0.935	0.921	0.963^3^	0.976^3^
- AUROC good total QoC score—relative	0.914	0.881	0.925^3^	0.935^3^
- AUROC poor total QoC score—relative	0.906	0.913	0.927^3^	0.940^3^ ^,^ ^4^
- OR good total QoC score—absolute (p-value)	68.92^5^ (p<0.001)	26.01 (p<0.001)	50.315 (p<0.001)	51.33 (p<0.001)
- OR good total QoC score—relative (p-value)	19.71^5^ (p<0.001)	13.49 (p<0.001)	40.35^5^ (p<0.001)	34.08^5^ (p<0.001)
- OR poor total QoC score—relative (p-value)	0.083 (p<0.001)	0.078 (p<0.001)	0.048^5^ (p<0.001)	0.029^5^ (p<0.001)
Range of performance frequency				
- # of items performed in <30% of cases	2	1	1	3
- # of items performed in <40% of cases	4	3	3	5
- # of items performed in >90% of cases	5	6	6	3
Distribution of index score				
- Coefficient of variation	23.08	21.40	21.90	28.52
- % of deliveries with minimum index score	0.00%	0.00%	0.00%	0.35%
- % of deliveries with maximum index score	0.35%	1.06%	1.06%	0.71%

^1^Coefficients and ORs are based on standardized index scores to enable comparison across indices with different numbers of items.

^2^Significant difference from coefficient for 3+ index (based on likelihood ratio test).

^3^Significant difference from AUC for 3+ index (Index B)

^4^Significant difference from AUC for preliminary QoPIIPC index (Index A)

^5^Significant difference from OR for 3+ index (based on likelihood ratio test).

### Validation results and recommended QoPIIPC index

Based on its relative performance on validation benchmarks, the second constructed index (Index G) was considered the optimal measure of QoPIIPC. The items in this index are listed in Table 11 and represent 4 of the 5 consensus QoPIIPC dimensions: technical quality, interpersonal care, screening and monitoring, and infection prevention/control. No items represent the avoidance of harmful/non-indicated interventions. This index covers intrapartum care, the immediate postpartum period, and ENC.

**Table 11:** Items in the recommended QoPIIPC index.

**Initial client assessment and examination**
Checks woman's HIV status (checks chart or asks woman) and/or offers woman HIV test
Asks whether woman has experienced headaches or blurred vision
Asks whether woman has experienced vaginal bleeding
Takes blood pressure
Takes pulse
Washes his/her hand before any examination
Wears high-level disinfected or sterile gloves for vaginal examination
**First stage of labor**
At least once, explains what will happen in labor to the woman and/or her support person
Prepares uterotonic drug to use for AMTSL
Uses partograph (during labor)
Self-inflating ventilation bag (500mL) and face masks (size 0 and size 1) are laid out and ready for use for neonatal resuscitation
**Second and third stage of labor**
Correctly administers uterotonic (timing, dose, route)
Assesses completeness of placenta and membranes
Assesses for perineal and vaginal lacerations
**Immediate newborn and postpartum care**
Immediately dries baby with towel
Places newborn on mother’s abdomen skin-to-skin
Ties or clamps cord when pulsations stop, or by 2–3 minutes after birth (not immediately after birth)
Takes mother's vital signs 15 minutes after birth
Palpates uterus 15 minutes after birth
Assists mother to initiate breastfeeding within one hour

The recommended QoPIIPC index score showed a statistically significant relation with the total QoC performance score across countries, with an increase of 2.24 to 2.77 points in the total QoC score for each one-point increase in the index score. This suggests that performing one additional intervention in the QoPIIPC index was associated with performance of multiple additional evidence-informed interventions during L&D care.

An increase in the recommended QoPIIPC index score also showed a statistically significant increase in the odds of being in the absolute and relative good performance categories for the total QoC score across countries. An increase in the recommended QoPIIPC index score was associated with a significant decrease in the odds of being in the poor performance category for the total QoC score (see Table 10 for illustrative results from Tanzania Round 1).

**Fig 2:**
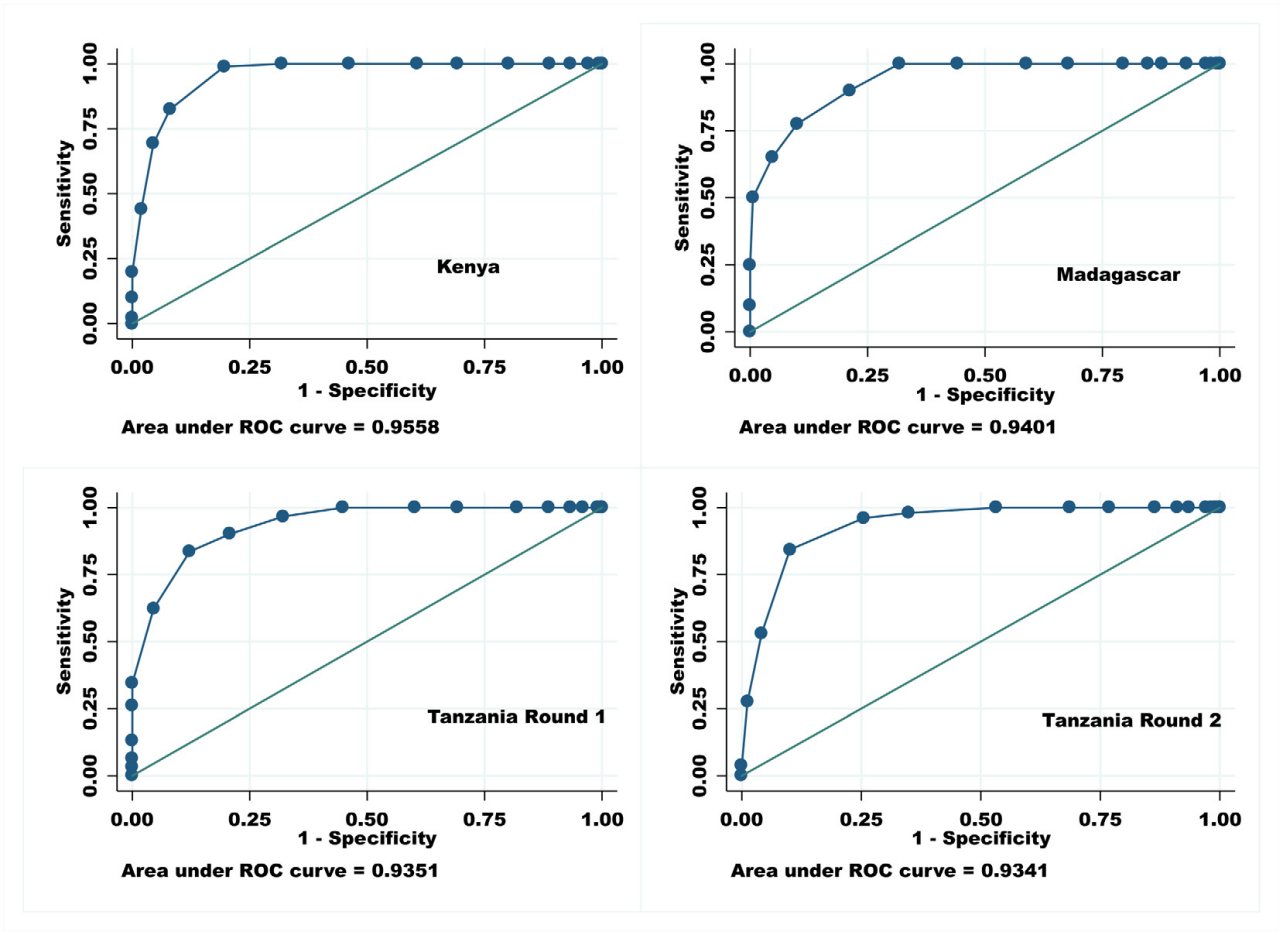
AUROCs (discrimination of good total quality score (top 25%)): Recommended QoPIIPC index.

The recommended QoPIIPC index showed excellent ability to identify absolute and relative good performance and relative poor performance across countries (Table 10). AUROCs ranged from 0.941 to 0.957 in Kenya, from 0.940 to 0.972 in Madagascar, from 0.935 to 0.976 in Tanzania Round 1, and from 0.918 to 0.934 in Tanzania Round 2. Fig 2 illustrates the AUROCs across countries for classification of delivery cases into the relative good performance group (the top 25% of total QoC scores). For example, if two deliveries were drawn from the sample at random in Madagascar, the recommended QoPIIPC index would correctly classify QoC in over 94% of these pairs; the case with the higher index score would be in the relative good performance group.

**Fig 3:**
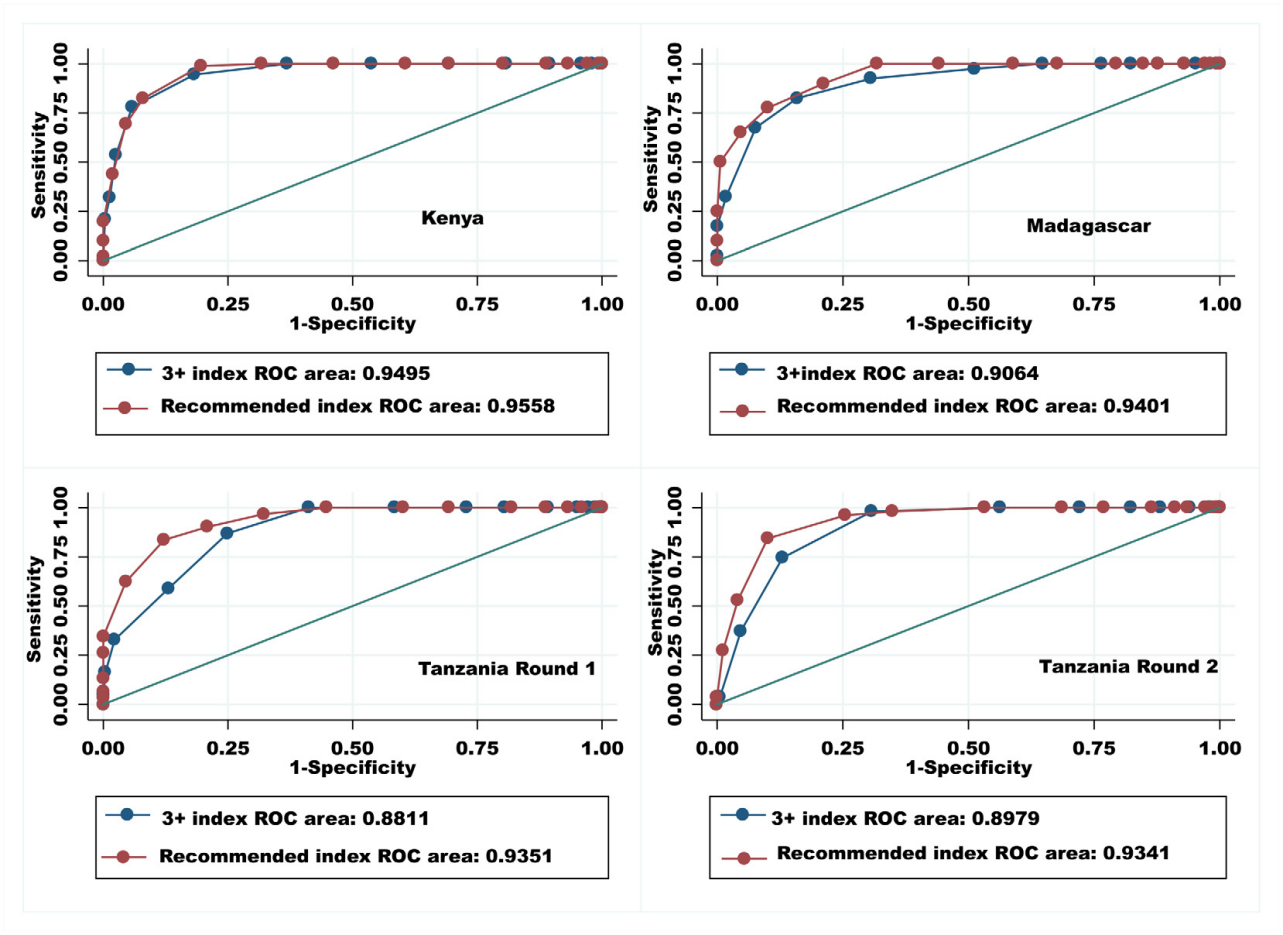
AUROCs (discrimination of good total quality score (top 25%)): Comparison of the 3+ index and the recommended index.

Comparison of AUROCs indicated that the recommended QoPIIPC index was often better able to classify deliveries into the poorly and well-performed delivery categories than the reference indices. Comparisons to Index B (the 3+ index) are illustrated in Fig 3. Similarly, likelihood ratio tests suggested that the recommended index fit the total QoC score data better in linear and logistic regressions than both reference indices (see Table 10 for illustrative results).

**Fig 4:**
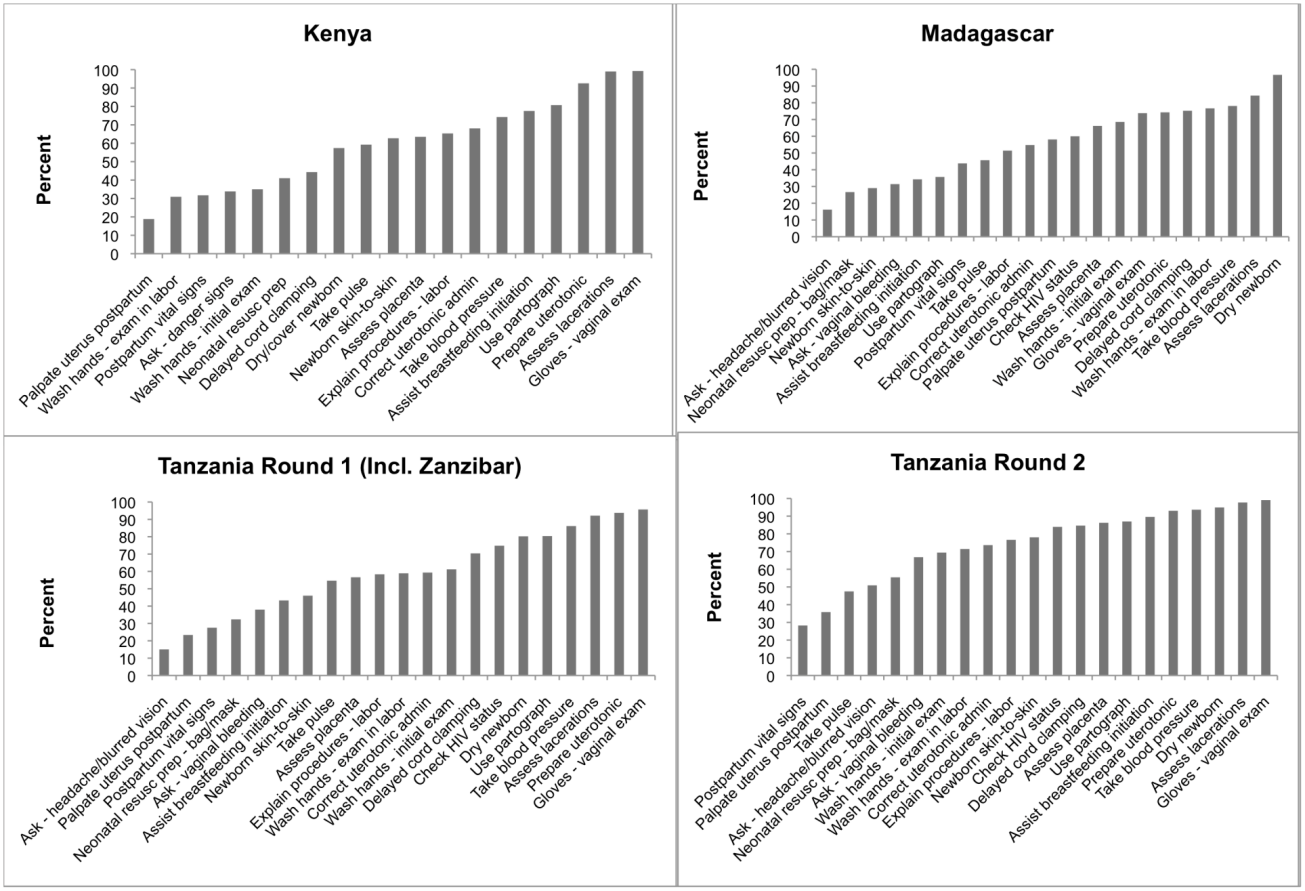
Frequency of performance of items in recommended QoPIIPC index across countries.

The frequency with which items in the recommended QoPIIPC index were performed varied across countries. The graphs in Fig 4 illustrate the spread of frequencies in each country, ranging from the least-performed to the most-performed item. The recommended index had the largest coefficient of variation relative to other potential QoPIIPC indices across all countries. No QoPIIPC index showed evidence of ceiling or floor effects.

Table 12 provides the predicted probabilities of being in the relative (top 25% of the total QoC score distribution) and absolute (≥80% of possible indicators performed correctly) good performance categories at different levels of the recommended QoPIIPC index score, using Tanzania Round 1 data. At the mean index score (12.12), the probability of being in the relative good performance group is only 4%. There is, however, a substantial increase in the likelihood of good performance with each one-point increase in the index score above the mean. A similar pattern is evident in the predicted probability of absolute good performance; because few (<3%) deliveries were in this category, the probabilities at each level of the index score remain very low.

**Table 12:** Predicted probabilities of good performance at different scores on recommended QoPIIPC index using Tanzania Round 1 (incl. Zanzibar) data.

Recommended QoPIIPC index (% frequency) n = 282	Predicted probability (CI) of good performance—relative^1^	Predicted probability (CI) of good performance—absolute^2^
0 (0.35%)	<0.001	<0.001
1 (0.00%)	<0.001	<0.001
2 (0.00%)	<0.001	<0.001
3 (0.35%)	<0.001	<0.001
5 (2.48%)	<0.001	<0.001
6 (2.13%)	<0.001 [<0.001–0.001]	<0.001
7 (3.55%)	<0.001 [<0.001–0.002]	<0.001 [<0.001–0.001]
8 (5.32%)	0.001 [<0.001–0.004]	<0.001 [<0.001–0.001]
9 (9.93%)	0.002 [<0.001–0.001]	<0.001 [<0.001–0.002]
10 (7.09%)	0.005 [0.001–0.020]	<0.001 [<0.001–0.003]
11 (12.06%)	0.013 [0.004–0.040]	<0.001 [<0.001–0.005]
**12 (10.64%)** ^3^	**0.036 [0.015–0.082]**	**<0.001 [<0.001–0.009]**
13 (10.28%)	0.093 [0.051–0.164]	0.001 [<0.001–0.016]
14 (8.16%)	0.222 [0.152–0.312]	0.002 [<0.001–0.029]
15 (10.64%)	0.441 [0.342–0.546]	0.008 [0.001–0.053]
16 (9.57%)	0.687 [0.559–0.792]	0.025 [0.006–0.099]
17 (1.77%)	0.859 [0.739–0.929]	0.075 [0.027–0.193]
18 (2.84%)	0.944 [0.859–0.979]	0.203 [0.089–0.397]
19 (1.42%)	0.979 [0.929–0.994]	0.443 [0.202–0.714]
20 (0.71%)	0.992 [0.965–0.998]	0.713 [0.348–0.920]
21 (0.71%)	0.997 [0.982–1.00]	0.886 [0.508–0.983]

^1^Relative good performance is defined as being in the top 25% of the total QoC score distribution.

^2^ Absolute good performance is defined as performing ≥80% of all observed routine L&D actions correctly; 2.84% of deliveries demonstrated absolute good performance

^3^ Mean = 12.12, median = 12

## Discussion and Conclusions

Through consensus building with MNC experts, this study identified five key dimensions of the quality of the process of intrapartum/immediate postpartum care (QoPIIPC): technical quality, interpersonal care, screening and monitoring, infection prevention/control, and avoidance of harmful/non-indicated interventions. Expert ratings of items representing these dimensions resulted in several potential indices to measure QoPIIPC. Comparative face, content, and criterion validation of the candidates resulted in selection of a 20-item measure with good ability to discriminate between poorly and well-performed deliveries. The recommended index includes items from 4 of the 5 consensus dimensions of QoPIIPC and covers intrapartum care, immediate postpartum care, and ENC.

### Limitations and strengths

This study had several limitations. First, study samples were limited to relatively high-volume facilities in each country and the lack of representative random sampling in each country may affect generalizability. However, given the effort involved in observing childbirth even with a reduced set of indicators, it may be appropriate to limit use of the proposed index to higher-volume facilities, for example those with at least two deliveries per day. Therefore, the study samples may adequately represent the likely context of future use of the QoPIIPC index.

An additional limitation is the limited number of respondents involved in the modified Delphi process to identify consensus dimensions of QoPIIPC. This reflects resource and time constraints; however, a larger and more diverse set of experts (n = 32) participated in the rating of potential items representing QoPIIPC, and indices combining highly-rated items were evaluated through the specified validation domains rather than exclusively through an expert process.

Another study limitation is that delivery care could not be evaluated in the QoC Assessments or using the QoPIIPC index proposed here without providers being aware of the observation. There is therefore the possibility of a Hawthorne effect, improving observed QoC beyond what is normally provided. However, providers cannot deliver interventions they do not know, and the relative low performance of many essential interventions in the observed deliveries indicates that any Hawthorne effect may be limited.

A final potential limitation is that the quality measure developed through this analysis is limited to assessment of routine care that should be provided to all women. As a result, it does not address important procedures and interventions that are required in subgroups of women (e.g., those who are HIV-positive) or women who develop complications. However, this focus on routine care enables the QoPIIPC index to provide information that complements what is frequently emphasized in current quality measures; this is discussed further below.

This study has a number of important strengths. The QoC Assessment samples covered a wide range of health facilities from university hospitals to rural health centers, which may contribute to generalizeablity, despite the lack of representative random sampling.

Additionally, the content validity of a measure depends in part on the comprehensiveness of the starting item pool. The item pool in the MCHIP QoC Assessment L&D checklist was based on compilation of evidence-informed interventions and program learning in maternal mortality reduction, providing a strong foundation for the final measure.

Much past research on the quality of obstetric care processes has relied on maternity registers and other routine data sources that may be incomplete, completed post facto, or not include measures of interest. This study is one of the few in developing countries with data on actual observations of labor and delivery. Observations may provide improvements in accuracy and specificity that counter disadvantages in terms of a Hawthorne effect [45, 47–48, 73]. Many recent studies identifying important indicators of QoC during the L&D period have been limited to expert surveys and literature reviews [74–75]. This study is unusual in selecting quality measures of L&D QoC in developing countries through both a Delphi process and validation with empirical data. Previous research suggests that quality measures based only on expert opinion may have weaknesses that can be addressed through empirical validation [44].

### Excluded and included items in the recommended QoPIIPC index

The recommended QoPIIPC index may be a robust quality measurement tool in a context of rapid change in QoC. As care quality continues to improve in facilities in sub-Saharan Africa, it may be important to focus rapid assessment on care processes that are easy to observe, not frequently performed (i.e., more useful in discriminating good care) and directly tied to adverse maternal/neonatal outcomes. The recommended QoPIIPC index reflects these priorities. Three items included in the reference indices and first QoPIIPC index constructed with expert feedback (index F), but removed from the recommended QoPIIPC index (Index G), are vaginal examination, abdominal examination, and cutting the umbilical cord with a clean instrument. Each represents an important care process but has limitations as an indicator of quality. Vaginal examination and safe cord cutting were almost universally performed across observed deliveries in the QoC Assessment surveys and may not be informative in distinguishing good and poor care. Additionally, it can be difficult for an observer to determine whether examinations include clinically important actions (such as assessment of cervical dilation and fetal presentation in vaginal examinations).

The three items in the recommended QoPIIPC index replacing the omitted items are: asking the woman whether she experienced headaches or blurred vision; palpating the uterus 15 minutes after delivery, and placing the newborn on the mother’s abdomen skin-to-skin immediately after birth. These items were performed less frequently in observed deliveries and judged by the MNC expert group to be more relevant to preventing adverse outcomes or responding quickly to danger signs.

These exclusions and inclusions may have contributed to the relatively stronger performance of the recommended QoPIIPC index on validation benchmarks.

### Program implications

The proposed QoPIIPC index has several attributes that address needs and constraints in low-resource settings. By condensing a much longer tool to 20 items, the index may make quality assessment less costly in time and human resources. Also, the recommended index and the original QoC surveys examine both maternal and neonatal care processes. Integrating assessment of care for the mother-newborn dyad is essential given limited resources for supervision in most developing countries and the fact that the same provider is often responsible for both the mother and newborn [76]. Finally, by focusing on routine care processes rather than clinical outcomes or complications, the index provides a quality measure that can be used to compare facilities without requiring adjustment for patient mix or disaggregating whether complications arose at the facility or at another site (e.g., patient’s home, referring facility) [77–79]. This simplification may ease planning of appropriate quality improvement (QI) efforts by supervisors who do not have the resources or data to conduct such analyses. Additionally, comparability of clinical indicators across sites is recommended in studies of quality assessment approaches [80–81].

The focus on routine care suggests another attribute of this new index that complements existing quality assessment tools. The most widely used indicators assessing maternal health programs in developing countries are the UN process indicators, which target EmONC [42, 82]. While there is no question of the importance of emergency care in preventing maternal and neonatal mortality, reliance on EmONC measures limits QoC assessment, particularly in smaller facilities where complications occur less frequently. Assessing quality exclusively through EmONC may also distort perceptions about overall service quality, as it does not evaluate provision of evidence-informed interventions shown to reduce the incidence of complications or timely recognition and management of danger signs before serious complications arise.

Notably, most of the UN process indicators focus on availability, utilization, and recent performance of service rather than QoC. The authors of the UN process indicators identify the case fatality rate as the sole quality indicator [42, 82]. The challenges of making inferences about quality based on case fatality rate, particularly without large sample sizes, have been noted [83]. The QoPIIPC index may provide programs with useful information in tandem with existing tools like the UN process indicators.

Many programs and implementation research studies continue to rely on service utilization measures that are not informative about QoC or clinical outcomes. A recent review examining MNC QI found that half of the included studies reported service utilization as their only outcome measure to assess the impact of QI strategies [84]. The application of tools like the QoPIIPC index may contribute to better information about MNC initiatives and the overall content of facility-based L&D care. The QoPIIPC index may have several potential programmatic uses, including baseline and ongoing assessments of QoPIIPC at the facility and district level as well as verification for ongoing QI or performance-based financing processes [85–87]. Rowe proposed the use of ongoing rapid assessments from household surveys and care observation to complement record review in monitoring the quality of L&D care [88]. The QoPIIPC index may be an appropriate tool for such repeated quality assessments.

It is important to note that the QoPIIPC index is not a job aid or tool to ensure that providers implement all essential or appropriate interventions; it is, rather, a selection of highly informative items to enable rapid QoC assessment. Tools such as the Safe Birth Checklist or the Standards-based Management and Recognition process may be more suited for clinical support and comprehensive QI efforts [42, 85].

### Research implications

A valid measure of QoPIIPC may facilitate future research on the determinants of good quality facility-based MNC and the effectiveness of different QI approaches. Although the results reported here suggest that the proposed index can provide a meaningful measure of the quality of intrapartum and immediate postpartum care, additional research is required about its feasibility, reliability, and perception by end users. Information can be gained through piloting the index in sub-Saharan African facilities by district-level supervisors and experienced clinical observers.

The strong performance of the QoPIIPC index on the validation domains, as a measure of overall QoC, is based on observation of all included items across the time from intake through the immediate postpartum period. While the index is substantially shorter than most existing tools for observation-based assessment of QoC in maternal and neonatal services, it continues to face challenges common to all observation of L&D—the timing of an obstetric episode of care is unpredictable and the length of active labor is frequently very long. As a result, supervisors and other potential users outside the research setting may be discouraged from using the index on an ongoing basis; potential users may wish to revert to records-based QoC assessment of the QoPIIPC index items or to base QoC assessment on observation of just some items in the index. While it may be possible to adapt the proposed index to retrospective, record-based usage, the disadvantages are outlined above. Although the QoPIIPC index has been validated only in the context of observation of complete episodes of L&D care, alternatives should be explored and may be necessary depending on the settings of use. Notably, 65% (13 of the 20) of the items comprising the proposed QoPIIPC index can be assessed at delivery or in the immediate intrapartum period. Using these items alone would reduce the content validity of the index (e.g., excluding most indicators related to screening). A shortened index may, nonetheless, be a useful alternative when there are limited resources for supervision. The relative validity and performance of a shortened QoPIIPC index focusing on the time of delivery warrants further evaluation.

## Conclusions

Currently, knowledge about the quality of L&D care processes in developing country settings is far too limited. Assessment of these processes is difficult with current tools, and a focus on EmONC and adverse event reviews limits understanding about coverage of routine interventions that can prevent complications and promote their early recognition and management. As financial incentives for women, performance-based financing for providers and facilities, removal of user fees, and other trends increase the proportion of women delivering in facilities, it is essential that these facilities provide quality care to women and newborns.

The global community has recognized the importance of QoC in achieving further reductions in maternal and neonatal mortality and morbidity and the need for valid ways to assess care quality. The index reported here provides a condensed, validated set of items that can be used to evaluate routine intrapartum and immediate postpartum care more easily using clinical observation. The availability of such a tool may improve knowledge about the quality of facility-based care for mothers and newborns in sub-Saharan Africa and other developing countries, and help programs target their efforts to improve quality.

## Supporting Information

S1 Checklist MCHIP QOC Assessment Tool 5—Observation of labor and delivery and newborn care checklist. (XLSX)

S1 Table Delphi group ratings of potential QoPIIPC dimensions (listed alphabetically). (DOCX)

S2 Table Performance of potential QoPIIPC index items across QoC Assessment countries. (DOCX)
