# Supplementary material for: Development and Validation of an Index to Measure the Quality of Facility-Based Labor and Delivery Care Processes in Sub-Saharan Africa
Source: PLoS One. 2015 Jun 24;10(6):e0129491. doi: 10.1371/journal.pone.0129491 (PMC4479466; doi:10.1371/journal.pone.0129491)
Supplement: S1 Table — (DOCX) [file pone.0129491.s002.docx]

**S1 Table. Delphi group ratings of potential QoPIIPC dimensions (listed alphabetically)**

| **Potential Dimension** | **Mean Importance Score**^1^ (n=7) | **Mean Uniqueness Score**^2^ (n=7) |
| --- | --- | --- |
| Action readiness | 3.0 | 2.57 |
| Avoidance of harmful/non-indicated practices | 2.86 | 2.29 |
| Infection prevention/control | 2.86 | 2.29 |
| Interpersonal | 2.86 | 2.57 |
| Monitoring | 2.86 | 2.14 |
| Record-keeping | 2.57 | 2.18 |
| Screening | 2.86 | 2.29 |
| Technical | 3.0 | 2.43 |

^1^Maximum importance score = 4.0

^2^Maximum uniqueness score = 3.0
