# Supplementary material for: Development and Validation of an Index to Measure the Quality of Facility-Based Labor and Delivery Care Processes in Sub-Saharan Africa
Source: PLoS One. 2015 Jun 24;10(6):e0129491. doi: 10.1371/journal.pone.0129491 (PMC4479466; doi:10.1371/journal.pone.0129491)
Supplement: S2 Table — (DOCX) [file pone.0129491.s003.docx]

**S2 Table. Performance of potential QoPIIPC index items across QoC Assessment countries**

| Variable | Proportion of deliveries in which indicator was correctly performed (95% confidence interval) | | | |
| --- | --- | --- | --- | --- |
|  | Kenya | Madagascar | Tanzania Round 1 | Tanzania Round 2 |
| Checks woman's HIV status (checks chart or asks woman) and/or offers woman HIV test^1^ | N/A | 0.60 (0.53 - 0.67) | 0.75 (0.70 - 0.80) | 0.84 (0.79 - 0.89) |
| Asks about any danger signs in pregnancy^2^ | 0.33 (0.29 - 0.38) | N/A | N/A | N/A |
| Asks whether woman has experienced fever | N/A | 0.21 (0.15 - 0.27) | 0.11 (0.07 - 0.15) | 0.34 (0.28 - 0.41) |
| Asks whether woman has experienced convulsions or loss of consciousness | N/A | 0.09 (0.05 - 0.13) | 0.08 (0.04 -0.11) | 0.21 (0.15 - 0.26) |
| Asks whether has experienced headaches or blurred vision | N/A | 0.16 (0.11 - 0.21) | 0.15 (0.11 - 0.19) | 0.51 (0.44 - 0.58) |
| Asks whether woman has experienced vaginal bleeding | N/A | 0.31 (0.25 - 0.38) | 0.38 (0.32 - 0.44) | 0.67 (0.61 - 0.73) |
| Takes temperature | 0.43 (0.38 - 0.48) | 0.47 (0.40 - 0.54) | 0.42 (0.36 - 0.48) | 0.33 (0.27 - 0.39) |
| Takes pulse | 0.59 (0.54 - 0.64) | 0.46 (0.39 - 0.53) | 0.55 (0.49 - 0.61) | 0.47 (0.41 - 0.54) |
| Takes blood pressure | 0.74 (0.70 - 0.79) | 0.78 (0.72 - 0.84) | 0.86 (0.82 - 0.90) | 0.94 (0.90 - 0.97) |
| Tests urine for presence of protein | 0.11 (0.08 - 0.14) | 0.04 (.01 - 0.06) | 0.06 (0.03 - 0.09) | 0.08 (0 .04 - 0.11) |
| Washes his/her hand before initial examination | 0.35 (0.30 - 0.40) | 0.69 (0.62 - 0.75) | 0.61 (0.55 - 0.67) | 0.69 (0.63 - 0.76) |
| Washes his/her hand before examination in labor | 0.31 (0.26 - 0.35) | 0.77 (0.71 - 0.82) | .59 (0.53 - 0.65) | 0.71 (0.65 - 0.78) |
| Abdominal examination (fetal presentation & fetal heart rate) | 0.97 (0.95 - 0.99) | 0.90 (0.85 - 0.94) | 0.91 (0.88 - 0.95) | 0.75 (0.69 - 0.80) |
| Vaginal examination (cervical dilation; fetal descent, position, membranes, meconium) | 0.99 (0.98 - 1.00) | 0.98 (0.96 - 1.00) | 0.99 (0.98 - 1.00) | 0.99 (0.98 - 1.00) |
| Wears high-level disinfected or sterile gloves for vaginal examination | 0.99 (0.98 - 1.00) | 0.74 (0.68 - 0.80) | 0.96 (0.93 - 0.98) | 0.99 (0.97 - 1.00) |
| At least once, explains what will happen in labor to the woman and/or her support person | 0.65 (0.61 - 0.70) | 0.51 (0.45 -0.58) | 0.58 (0.52 - 0.64) | 0.77 (0.71 - 0.82) |
| Uses partograph | 0.81 (0.77 - 0.85) | 0.36 (0.29 - 0.42) | 0.80 (0.76- 0.85) | 0.87 (0.82 - 0.92) |
| Prepares uterotonic drug to use for AMTSL | 0.93 (0.90 - 0.95) | 0.74 (0.68 - 0.80) | 0.94 (0.91 - 0.97) | 0.93 (0.90 - 0.96) |
| Prepares items for neonatal resuscitation | 0.41 (0.36 - 0.46) | N/A | N/A | N/A |
| Self-inflating ventilation bag (500mL) and face masks (size 0 and size 1) are laid out and ready for use for neonatal resuscitation^3^ | N/A | 0.27 (0.21 - 0.33) | 0.32 (0.27 - 0.38) | 0.55 (0.49 - 0.62) |
| At least 3 cloths/blankets (1 to dry, 1 to cover, 1 to elevate shoulders) are laid out and ready for use for neonatal resuscitation | N/A | 0.79 (0.73 - 0.84) | 0.39 (0.33 - 0.45) | 0.71 (0.65 - 0.77) |
| Puts on clean protective clothing in preparation for birth that protects face, hands, and body from contact with body fluids | 0.68 (0.63 - 0.72) | 0.60 (0.53 - 0.66) | 0.37 (0.31 - 0.43) | 0.50 0.44 - 0.57) |
| As baby's head is delivered, supports perineum | 0.97 (0.95 - 0.98) | 0.77 (0.71 - 0.83) | 0.87 (0.83 - 0.91) | 0.89 (0.84 - 0.93) |
| Correctly administers uterotonic (timing, dose, route) | 0.68 0.63 - 0.73) | 0.55 (0.48 - 0.62) | 0.59 (0.53 - 0.65) | 0.74 (0.68 - 0.80) |
| Performs uterine massage immediately after delivery of placenta | 0.87 (0.83 - 0.90) | 0.55 (0.48 - 0.62) | 0.55 (0.49- 0.60) | 0.91 (0.88 - 0.95) |
| Assesses for perineal and vaginal lacerations | 0.99 (0.98 - 1.00) | 0.84 (0.79 - 0.89) | 0.92 (0.89 - 0.95) | 0.98 (0.96 - 1.00) |
| Assesses completeness of placenta and membranes | 0.64 (0.59 - 0.68) | 0.66 (0.60 - 0.73) | 0.57 (0.51 - 0.62) | 0.86 (0.82 - 0.91) |
| Immediately dries baby with towel | 0.57 (0.53 - 0.62) | 0.97 (0.94 - 0.99) | 0.80 (0.75 - 0.85) | 0.95 (0.92 - 0.98) |
| Discards wet towel and covers with dry towel^4^ | N/A | 0.81 (0.76- 0.86) | 0.79 (0.74- 0.83) | 0.95 (0.93 -0.98) |
| Places newborn on mother’s abdomen skin-to-skin | 0.63 (0.58 - 0.67) | 0.29 (0.23- 0.35) | 0.46 (0.40 - 0.52) | 0.78 (0.72 - 0.84) |
| Ties or clamps cord when pulsations stop, or by 2 - 3 minutes after birth (not immediately after birth) | 0.44 (0.39 - 0.49) | 0.75 (0.69 -0.81) | 0.70 (0.65- 0.76) | 0.85 (0.80 - 0.90) |
| Cuts cord with clean blade | 0.96 (0.94 - 0.98) | 0.75 (0.69 - 0.81) | 0.99 (0.97 - 1.00) | 0.99 (0.98 - 1.00) |
| Disposes of all sharps in puncture-proof container immediately after use | 0.96 (0.94 - 0.98) | 0.94 (0.91 - 0.97) | 0.92 (0.89 - 0.95) | 0.98 (0.96 - 1.00) |
| Takes mother's vital signs 15 minutes after birth | 0.32 (0.27 - 0.36) | 0.44 (0.37 - 0.51) | 0.28 (0.22 - 0.33) | 0.28 (0.22 - 0.34) |
| Palpates uterus 15 minutes after delivery of placenta | 0.19 (0.15 - 0.23) | 0.58 (0.51 - 0.65) | 0.23 (0.18 - 0.28) | 0.36 (0.29 - 0.42) |
| Assists mother to initiate breastfeeding within one hour | 0.78 (0.73 - 0.82) | 0.34 (0.28 - 0.41) | 0.43 (0.37 - 0.49) | 0.90 (0.85 - 0.94) |

^1^The HIV/AIDS question was not asked in Kenya

^2^Separate questions about danger signs were not asked in Kenya.

^3^Separate questions about neonatal resuscitation preparation items were not asked in Kenya

^4^Questions about immediate drying, discarding of wet towel, and wrapping in dry towel were combined in Kenya.
